# **MBRA 3.0: integrating the mucus environment for advanced high-throughput** ***in vitro*** **intestinal microbiome modeling**

**DOI:** 10.1080/19490976.2026.2612804

**Published:** 2026-01-11

**Authors:** Maeva Duquesnoy, Benoit Chassaing

**Affiliations:** a Microbiome-Host Interactions, Institut Pasteur, Université Paris Cité, INSERM U1306, Paris, France

**Keywords:** Microbiota, mucus, *in vitro* model, MBRA 3.0, microbiome compartmentalization

## Abstract

The colonic mucus layer is a dynamic barrier that plays central roles in intestinal health, and recent studies highlight that it harbors a distinct and functionally critical microbial community. However, most *in vitro* gut models fail to recapitulate this mucosal niche, limiting mechanistic investigation of microbiota–mucus interactions. Here, we developed the MBRA 3.0 system, a next-generation chemostat engineered to integrate mucus-coated carriers and enable high-throughput dissection of spatial microbiome dynamics. Using fecal microbiota from eight human donors, we report that mucus addition does not impact total bacterial density but selectively shapes microbial community structure, metabolic output, and pro-inflammatory potential in a donor-dependent manner. Notably, MBRA 3.0 resolves stable, compositionally distinct mucus-associated and luminal communities, mirroring *in vivo* spatial heterogeneity. Integration of this mucosal niche also modulates short-chain fatty acid (SCFA) profiles and inflammatory signatures, highlighting the relevance of the spatial context for intestinal microbiota research. Hence, MBRA 3.0 offers a scalable and customizable platform to model mucus–microbiota interactions, advancing our understanding of gut ecology and supporting translational discovery in gastrointestinal health and disease.

## Introduction

The intestinal mucus layer serves as a crucial physical and biochemical barrier, maintaining gut health by controlling interactions between host cells and the luminal microbiota. In the colon, this barrier exhibits a sophisticated structure comprising two distinct layers: a dense, sterile inner layer firmly attached to the epithelium,^1^^,^^2^ providing direct protection from microbial invasion and chemical insults, and a looser, outer layer, colonized by specific microbial ecosystem.^3^ Recent research reported that communities inhabiting the mucus differ in both composition and function from those dwelling in the gut lumen.^4^^,^^5^ Particularly, bacteria equipped with carbohydrate-active enzymes (CAZymes) are enriched in the mucus compartment,^5^^,^^6^ supporting their ability to degrade and navigate the mucus structure.

Such stratified spatial organization sustains host defense and supports microbial diversity, as demonstrated in MUC2-deficient mice which develop spontaneous colitis due to the absence of a functional mucus barrier.^7^ Further evidence for the importance of this separation comes from studies of microbiota encroachment – colonization of the inner mucus layer by select microbial populations – which has recently emerged as a hallmark of several chronic diseases, including inflammatory bowel disease (IBD) and metabolic syndrome, in both mouse models and humans.^8-10^ Strikingly, encroachment not only correlates with disease but may act as a driver of pathology: in a recent study, Kordahi et al. reported that transferring the mucus-penetrating bacterial community from mouse models exhibiting microbiota encroachment into germ-free mice was sufficient to induce chronic inflammatory conditions.^11^^,^^12^ These findings underscore the critical role of maintaining spatial compartmentalization between the microbiota and the host mucosa in intestinal health.

Certain conditions, such as inflammatory bowel diseases (IBD) and exposure to environmental factors such as dietary emulsifiers, can erode the separation between the microbiota and the colonic epithelium, encouraging the accumulation of flagellated, mucus-penetrating bacteria that promote inflammation.^13^^,^^14^ Despite this significance, there is still a limited understanding of how the gut microbiota dynamically interact with mucus, mainly due to the lack of accessible *in vitro* models replicating these spatially distinct niches.

Based on these observations, a wide range of *in vitro* gut models has been developed to reproduce the complexity of the intestinal environment. Systems such as the SHIME,^15^ which recapitulates the entire gastrointestinal tract from the stomach to the distal colon, or the ARCOL,^16^ PolyFermS,^17^ and TNO's TIM-2^18^ models, which incorporate peristaltic movements, enable long-term cultivation of the intestinal microbiota under tightly controlled conditions. These platforms operate at relatively large working volumes, facilitating extensive downstream analyses and offering high reproducibility. However, these methods remain labor-intensive, technically demanding, and relatively low-throughput, limiting their capacity for parallel experimentation. To address these limitations, benchtop models such as the MiniColon Model (MiCoMo)^19^ and the MiniBioReactor Array (MBRA)^20^^,^^21^ have been introduced. Their compact format supports highly parallelized, customizable continuous-flow cultivation, thereby improving throughput and experimental flexibility. Importantly, none of these high-throughput systems incorporate a mucus component, while only larger, low-throughput platforms have been engineered to include this feature (such as the M-SHIME^22^ and M-ARCOL^23^ models). As a result, there remains a need for scalable systems capable of distinguishing luminal from mucus-associated communities and enabling targeted investigation of mucus–microbiota interactions. To overcome these limitations, we developed an adapted version of the MiniBioReactor Array (MBRA)^20^^,^^21^ system. While the recently developed MBRA 2.0 enabled investigation of the biofilm-forming population,^24^ it did not allow differentiation between luminal and mucus-associated communities or enable targeted study of mucus‒microbiota interactions. Here, we developed the MBRA 3.0 system, a refined version that incorporates mucus-coated carriers enabling direct comparison between luminal and mucosal microbiota within a high-throughput and parallelizable continuous framework.

To test our system performances, we cultured fecal microbiotas from eight human donors with and without mucus-coated carriers and longitudinally analyzed these community over a 10 d period. While total bacterial density and richness were largely unaffected by the presence of mucus, we importantly observed mucus-dependent shifts in community structure, allowing distinct separation between luminal and mucus-associated profiles, as well as alterations in short-chain fatty acid (SCFA) production and pro-inflammatory potential. Hence, our results confirm that the MBRA 3.0 system supports the stabilization of compositionally distinct mucus-associated and luminal communities, mirroring the spatial heterogeneity observed *in vivo*. The incorporation of a defined mucosal niche also modulates short-chain fatty acid (SCFA) profiles and inflammatory signatures, underscoring the central role played by mucus-associated populations in shaping microbiota function. Together, these considerations highlight the critical importance of integrating a mucus compartment into *in vitro* intestinal models to improve physiological relevance and suggest that the MBRA 3.0 provides a scalable and customizable platform to advance future research into microbiota–host interactions.

## Results

### Modifications of the MBRA *in vitro* microbiota modeling system to study mucus-associated microbiota

**Figure 1.**
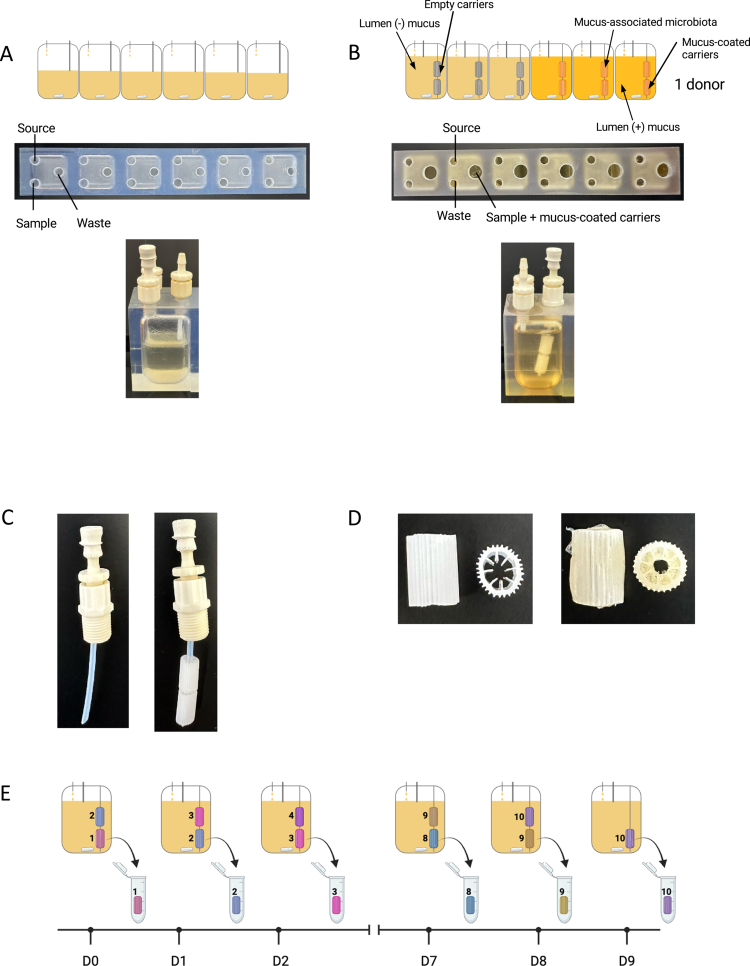
Modifications of the MBRA *in vitro* microbiota modeling system to study mucus-associated microbiota. A. Schematical representations and images of the original MBRA *in vitro* microbiota system used for luminal microbiota culture. B. Schematical representations and images of the MBRA 3.0 design to allow the insertion of mucus-coated carriers, enabling the coexistence of luminal and mucus-associated microbiota. C. Tubing and connector used to add mucus-coated carriers inside the bioreactors of the MBRA 3.0 system. D. Carriers used for mucus supplementation in the MBRA 3.0 system, with an empty carrier on the left and a mucus-loaded carrier on the right. E. Schematical representation of mucus-coated carriers' turnover and sampling overtime.

MBRA 3.0 is an advanced adaptation of the original high-throughput MBRA chemostat^25^ intended to more closely mimic distal colonic conditions by integrating a mucus phase. The original platform comprises four racks with six reactors per rack (25  mL total, 15  mL working volume), each with a distinct medium inlet, effluent port, and sampling hub^20^ (Figure 1A). To support mucus‒carrier integration in the MBRA 3.0 system, we modified the reactors by enlarging the sampling port in order to allow them to pass through mucus-coated carriers (Figure 1B,C). Furthermore, we shortened the effluent tubing, leading to an increased working volume to 20 mL, ensuring complete mucus carrier immersion (Figure 1B). Next, to mimic the mucus-associated microbial environment, mucus carriers were coated with a mucin–agar matrix prepared by combining sterile boiled water with 1% (w/v) agar to facilitate mucin adherence, and 5% (w/v) porcine gastric mucin (type II). Once homogenized, the matrix pH was adjusted to 6.8, then the sterile carriers were immersed in the hot mixture, air-dried under a safety cabinet and stored in a humid chamber at 4 °C until use (Figure 1D).

With our central goal of longitudinally modeling the mucus-associated microbial population together with repeated sampling, we decided to simultaneously use two mucus-carrier per MBRA chambers. At the time of inoculation, each reactor received two mucus-coated carriers, and in order to emulate intestinal mucus turnover, one of the two carriers was replaced daily with a sterile and freshly-coated carrier, resulting in an in-reactor lifespan of 48 h for each carrier (Figure 1E) together with constant presence of “mature” mucus-associated microbial ecosystem, the more recent carrier present at a given time being immerged for at least 24 h (Figure 1E).

### Bacterial density remains unchanged in MBRA 3.0 containing mucus-coated carrier

Prior studies have consistently reported that the MBRA system can sustain complex human fecal microbiotas in continuous culture for prolonged periods.^20^^,^^26^^,^^27^ Hence, we first investigated whether these structural modification of the original MBRA system, and/or the addition of mucus-coated carriers, could influence the stability or community structure of complex ecosystems. For this purpose, eight fecal donors were cultured in MBRA 3.0 reactors containing either uncoated or mucus-coated carriers, as presented Figure 1B. In the following sections, samples coming from reactors with uncoated carriers are referred as “***Lumen – Mucus***”, samples from reactors with mucus-coated carriers are referred to as “***Lumen + Mucus***”, and samples from mucus-coated carriers are referred to as “***Mucus-associated microbiota***”.

**Figure 2.**
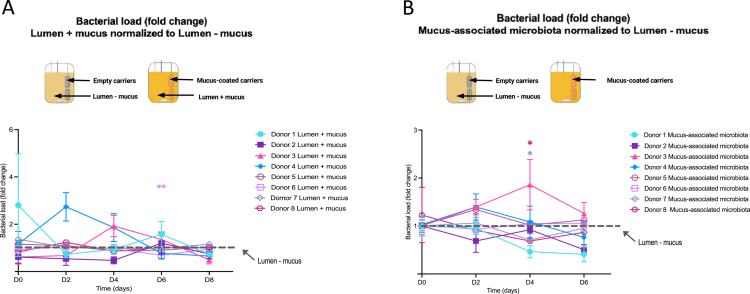
Quantification of the bacterial load in the lumen + mucus and mucus-associated microbiota Compared to lumen – mucus microbiota. A. Longitudinal quantification of the bacterial load in luminal microbiota from lumen + mucus compartments. The data were normalized to the lumen – mucus microbiota, which was defined as 1. The data are expressed as mean + /− S.E.M. Significance was assessed by two-way ANOVA and is indicated as follows: *p ≤ 0.05; **p ≤ 0.01. B. Longitudinal quantification of bacterial load in mucus carriers. The data were normalized to the lumen – mucus microbiota, which was defined as 1. Each line and symbol represent an individual donor (*N* = 8). The data are expressed as mean + /− S.E.M. Significance was assessed by two-way ANOVA and is indicated as follows: *p ≤ 0.05; **p ≤ 0.01.

We first performed longitudinal analysis of the total bacterial load using primers specific to the V4 region of the 16SrRNA gene and expressed the lumen bacterial load in mucus-containing chambers compared to lumen bacterial load in mucus-free chambers. These various time points were next combined in a single XY representation with two normalizations being applied: first, the bacterial load in the lumen–mucus samples was defined as 1 for each timepoint. Next, triplicate values of the lumen + mucus and mucus carriers samples were normalized to 1 at Day 0 (D0), which corresponds to the mucus carriers addition within the system. This analysis revealed that, in the MBRA 3.0 system, the introduction of mucus-coated carriers inside the bioreactors does not significantly alter the microbial density on the lumen side, as evidenced by comparable bacterial densities between conditions with and without mucus (Figure 2A, Figure S1A). Although select donors (#3 and #4) show a transient increase in bacterial density in the lumen + mucus compartment, which quickly returns to a level comparable to the lumen – mucus compartment.

We next analyzed the bacterial load in the mucus compartment itself by performing DNA extraction and 16S-based qPCR quantification of bacteria associated with the mucus-carrier. Such analysis, presented in Figure 2B and Figure S1A, highlighted the robust and stable colonization of the introduced mucus carriers without major donor-dependent effects. Altogether, these data suggest slight but non-significant modulation of luminal bacterial density when mucus was added to the MBRA system, together with a stable colonization of the introduced mucus niche.

### The addition of a mucus niche modestly impacts microbiota composition compared to inter-individual variability

Multiple studies have reported that specific members of the intestinal microbiota, particularly those inhabiting the upper mucus layer of the colon, possess the ability to degrade mucin and utilize it as an alternative energy source under conditions of limited carbohydrate availability, such as during dietary fiber deprivation.^28^^,^^29^ Moreover, some bacteria feed primarily on mucus from the mucus layer.^30-32^ Hence, we next examined the microbiota composition of individual donors in reactors with and without mucus, in order to determine whether specific microbial taxa exhibited a selective advantage in the presence of mucus.

**Figure 3.**
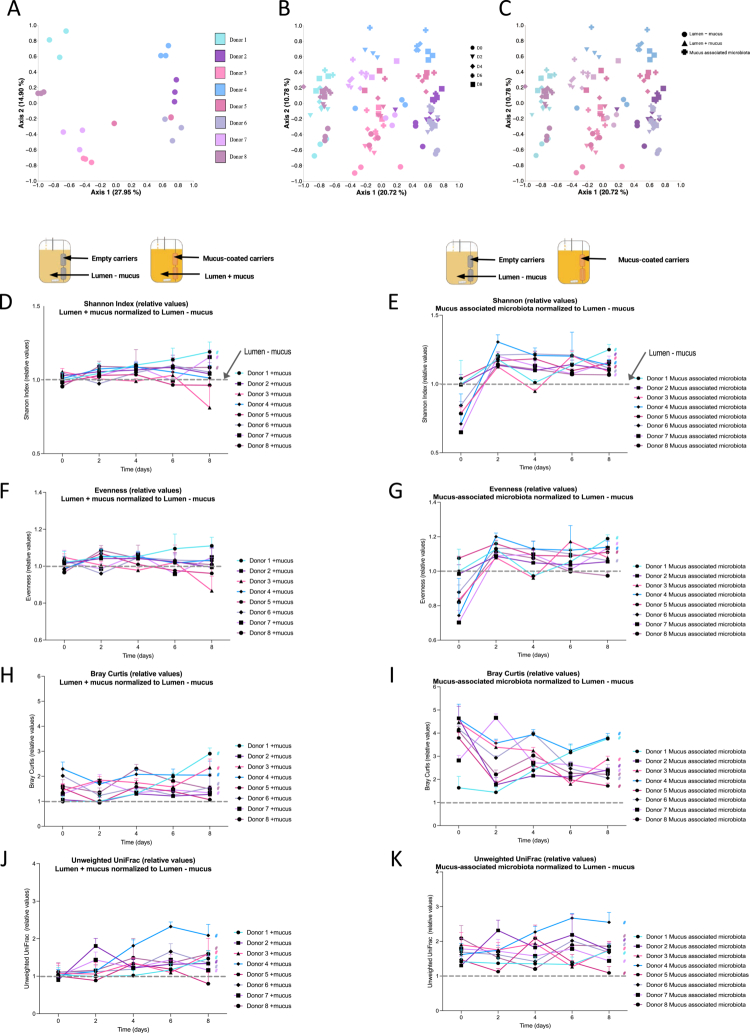
Temporal and inter-donor variation in the community structure and diversity of lumen + mucus and mucus-associated gut microbiota compared to lumen – mucus microbiota. A. Principal coordinates analysis (PCoA) of lumen – mucus microbiota for all donors (*N* = 8) at day 6, based on unweighted UniFrac distances. Each color represents 1 donor. B. PCoA of lumen – mucus microbiota for all donors (*N* = 8) and all timepoints (*n* = 6), based on unweighted UniFrac distances. Each color represents 1 donor and each symbol represents a different timepoint (circles = D0; triangles = D2; diamonds = D4; crosses = D6; squares = D8) C. PCoA of microbial communities from all donors (*N* = 8) at day 8, including lumen – mucus, lumen + mucus and mucus-associated microbiota, based on Unweighted UniFrac distances. Each color represents a donor and each symbol represents a condition (circles = lumen – mucus; triangles = lumen + mucus; crosses = mucus-associated microbiota). D–G. Longitudinal analysis of alpha diversity metrics (D,E, Shannon index; F,G, Evenness index) of lumen + mucus (D, F) and mucus-associated microbiota (mucus carriers; E, G). The data were normalized to the lumen – mucus microbiota, which was defined as 1. Each line color and symbol represents an individual donor (*N* = 8). The data are expressed as mean + /- S.E.M., and significance was assessed by two-way ANOVA and is indicated as follow: # *p* ≤ 0.05. H‒K. Longitudinal analysis of beta diversity metrics (H‒I, Bray‒Curtis distance; J‒K, unweighted UniFrac distance) of lumen + mucus (H, J) and mucus-associated microbiota (mucus-associated microbiota; I, K). The data were normalized to the lumen – mucus microbiota, which was defined as 1. Each line color and symbol represents an individual donor (*N* = 8). The data are expressed as mean + /- S.E.M. Significance was assessed by two-way ANOVA and is indicated as follow: # *p* ≤ 0.05.

Principal coordinate analysis based on the Unweighted UniFrac distance revealed that the microbiota composition of lumen – mucus samples, for each donor, was distinct and reproducibly maintained across the three parallel reactors collected at day 6 (Figure 3A). This finding aligns with some of our previous findings, with inter-individual variations in donor's microbiota being efficiently reproduced and maintained over time in the MBRA system.^26^^,^^27^^,^^33^^,^^34^ Moreover, further highlighting this point, we observed that the microbiota of a given donor remained more similar to its initial timepoint (baseline, D0) throughout the experiment (D2, D4, D6, and D8) compared to that of other donors over time (Figure 3B).

We next investigated the potential effect of adding a mucus niche in the MBRA 3.0 system on the luminal microbiota composition. For this purpose, we performed principal coordinate analysis based on the unweighted UniFrac distance using day 8 samples, including all 8 donors and all types of samples (lumen – mucus, lumen + mucus, mucus carriers). This analysis showed a strong donor-based clustering, with secondary clustering based on sample type (Figure 3C). Hence, these data importantly revealed that while the addition of a mucus niche allowed the MBRA 3.0 system to keep its ability to maintain inter-individual variations in microbiota composition, it also allows to cultivate different ecosystems driven by the presence of a mucus component, as further evidenced through longitudinal analysis of principal coordinate between lumen – mucus and lumen + mucus ecosystem (Figure S2).

We next performed alpha diversity analysis and observed no major differences in the Shannon and Evenness indices over time between the luminal microbiota in the presence versus in the absence of mucus, which was consistent with previous observations made using the MBRA system highlighting the relative resilience of the MBRA system for alpha diversity fluctuations (Figure 3D,F). When comparing microbial richness of the mucus-associated microbiota with that of the luminal microbiota (lumen – mucus), we observed a less diverse mucus-associated community at D0 (Figure 3E,G; Figure S1D,E). This observation appears to be consistent with the time required for the mucus layer to become fully colonized, together with the fact that various community members need a couple of days to establish stable populations within the MBRA system.^27^^,^^35^ For the subsequent timepoints, we observed an approximately 20% increase in mucus-associated microbiota richness compared to the luminal side (Figure 3E,G, Figure S1D,E; *p* ≤ 0,05), highlighting that mucus carriers are being colonized over time in the MBRA 3.0 system, with a microbial community of relatively similar richness but with compositional variations compared to the luminal ecosystem. Hence, to further investigate this notion, we next performed longitudinal microbiota composition analysis. Importantly, temporal differences were observed between the luminal microbiota in the presence versus in the absence of mucus, with the degree of variation differing between donors (Figure 3H,J). The microbiota composition of donors 1, 2, 3, 4, 6, and 7 were most affected by the presence of mucus, whereas those of donors 5 and 8 remained largely unchanged over time (*p* ≤ 0,05). These data highlight that the introduction of mucus carriers in the MBRA 3.0 system are sufficient to significantly impact the luminal microbiota composition in a donor-dependent but also in a mucus presence dependent manner.

**Figure 4.**
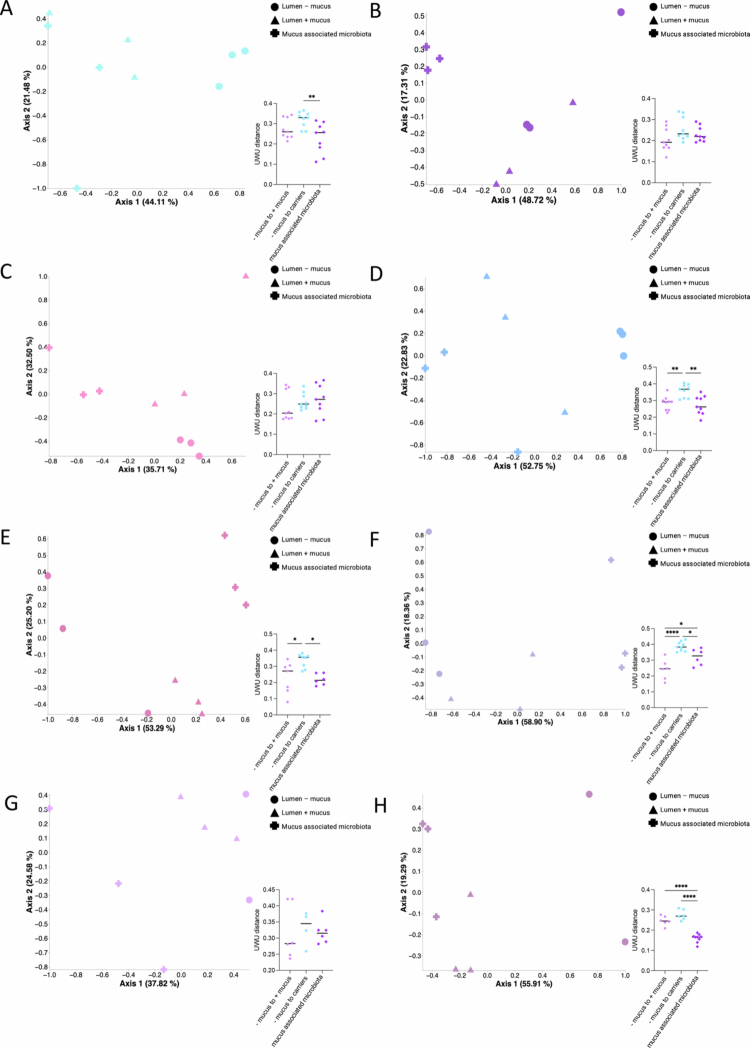
Intra-donor variations in microbiota composition between the lumen – mucus, lumen + mucus, and mucus-associated microbiota. A–H. PCoA of lumen – mucus, lumen + mucus and mucus-associated microbiota from each donor (*N* = 8) at day 8, based on unweighted UniFrac distances. Each symbol represents a different condition (circles = lumen – mucus; triangles = lumen + mucus; crosses = mucus-associated microbiota). For each PCoA plot, a grouped scatter dot plot represents the unweighted UniFrac distance between (*a*) lumen – mucus and lumen + mucus ecosystems (light purple); (*b*) lumen – mucus and mucus-associated ecosystems (light blue); and (*c*) lumen + mucus and mucus-associated ecosystems (dark purple). Data are expressed as mean + /− S.E.M. Significance was assessed by one-way ANOVA and is indicated as follow: *p ≤ 0.05; **p ≤ 0.01; ***p ≤ 0.001; ****p ≤ 0.0001.

Similar longitudinal analysis were subsequently performed on the mucus carrier-associated microbiota and compared to the luminal microbiota. This approach revealed that the composition of the mucus-associated microbiota strikingly differs from that of the luminal microbiota (+mucus) (Figure 3I,K). This was further evidenced when we performed principal coordinate analysis based on the unweighted UniFrac distance using day 8 samples, including all three types of samples (lumen – mucus, lumen + mucus, mucus-associated microbiota) with only 1 donor per plot (Figure 4), with the observation that while all the three sample types formed distinct clusters, lumen – mucus and lumen + mucus samples were closer compared to mucus-associated microbiota samples. This distinction was further supported by principal coordinate analyses based on multiple distance metrics (Bray–Curtis, weighted UniFrac, unweighted UniFrac, and Jaccard) performed on all samples collected throughout the experiment (Figure S3). Across all the metrics, the mucus-associated microbiota formed well-defined clusters that were clearly separated from both luminal conditions (Adonis2 *p* = 0.001). The effect size was highest for weighted UniFrac distances (R² = 0.16), indicating that the mucus matrix selected for shifts in abundant phylogenetic lineages, whereas Bray–Curtis, Jaccard, and unweighted UniFrac distances revealed additional contributions from rare taxa. PERMDISP tests showed no significant differences in dispersion for Bray–Curtis and weighted UniFrac distances, suggesting that separation is driven by compositional differences rather than differential variability, whereas Jaccard and unweighted UniFrac tests revealed modest dispersion differences (Figure S3).

To determine when these communities begin to diverge, we next performed time-resolved PCoA analyses using Bray–Curtis distances at each sampling day (D0, D2, D4, D6, D8; Figure S4). At baseline (D0), all the three sample types showed partial overlap but were already significantly separated (R² = 0.18, *p* = 0.001), which is consistent with the rapid establishment of distinct community structures upon inoculation. By D2 and D4, the separation between the mucus-associated microbiota and luminal samples became more pronounced (R² = 0.165–0.17, *p* = 0.001). At D6, the groups remained significantly distinct, although PERMDISP indicated a slight dispersion difference at this timepoint. By D8, the separation reached its highest effect size (R² = 0.193, *p* = 0.001), confirming that with continued cultivation, mucus-associated communities consistently diverge from luminal communities and maintain a stable, distinct compositional identity (Figure S4).

These data revealed that while the addition of a mucus niche allowed the MBRA 3.0 system to maintain its ability to maintain inter-individual variations in microbiota composition, it also allows to cultivate different ecosystems driven by the presence of a mucus component. Hence, the MBRA 3.0 system appears suitable for growing a specific mucus-associated microbial ecosystem, which is well aligned with previous pre-clinical and clinical findings that luminal and mucosal microbial communities exhibit distinct profiles.^4^^,^^36^^,^^37^

### The mucus niche modulates select microbiota members in the MBRA 3.0 system

**Figure 5.**
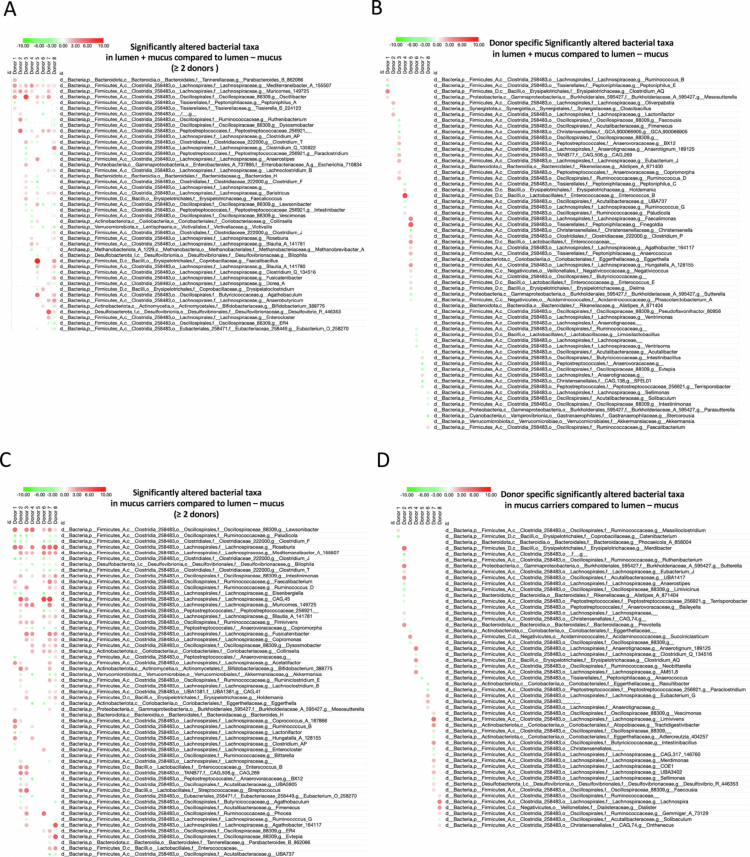
Microbial feature alterations in lumen + mucus and mucus-associated microbiota ecosystems compared to lumen – mucus. A. Heatmap of microbial features significantly altered in at least two donors between the lumen + mucus and lumen – mucus compartments. B. Heatmap of microbial features significantly altered in a single donor between the lumen + mucus and lumen – mucus compartments. C. Heatmap of microbial features significantly altered in at least two donors between mucus-associated and lumen – mucus compartments. D. Heatmap of microbial features significantly altered in a single donor between between mucus-associated and lumen – mucus compartments. Dots size and color represent the fold change in bacterial abundance relative to the lumen – mucus microbiota, with a minimal – 10 (green) to a maximal + 10 (red) fold change range, as assessed by MaAslin2.

Next, to delve deeper into mucus-induced microbiota modulation, we performed taxonomical analysis at the order level in stabilized MBRA chambers (day 8). This analysis, which revealed donor-dependent variations in microbial composition, also revealed shifts in the abundance of select luminal taxa between the three types of samples generated (lumen – mucus, lumen + mucus, mucus carriers). For example*, Lachnospirales* were consistently more abundant in the mucus compartment compared to the lumen (Figure S5). Hence, to identify microbial taxa significantly affected by the presence of mucus carriers, we next performed microbiome multivariable association with linear model (MaAsLin2) analysis. This revealed significant changes in the relative abundance of 45 luminal genera in at least two donors (Figure 5A), of 40 luminal genera unique to a given donors (Figure 5B), 57 mucus-associated genera in at least two donors (Figure 5C), and 51 mucus-associated genera unique to a given donors (Figure 5D). For example, the genus *Lawsonibacter*, a member of the *Firmicutes* phylum previously reported as the predominant member of the mucus-associated microbiota,^38^ harbored a strong increase in its relative abundance in the mucus niche compared to the luminal ecosystem in 6 donors (Figure 5C, *q* ≤ 0,05). Moreover, several microbial taxa with well-established roles in gut health respond selectively to mucin supplementation. *Roseburia* and *Faecalibacterium*, two major butyrate producers^39-42^ linked to the anti-inflammatory effects of the epithelial barrier support^43-47^ and found to be reduced under chronic inflammatory conditions,^48^ increased in abundance, with *Roseburia* enriched across multiple donors in both lumen + mucus and mucus carrier compartments (Figure 5A,C; qval < 0,0001) and *Faecalibacterium* increasing specifically in the mucus carriers (Figure 5C; qval < 0,0001). *Akkermansia muciniphila*, despite its known preference for the mucus niche^31^^,^^49^ and frequent association with metabolic health,^50-52^ decreased in abundance in mucus carriers (Figure 5C; qval < 0,0001) and was notably reduced in the lumen + mucus compartment of donor 8 (Figure 5B; qval < 0,0001). *Bilophila*, a bacterium associated with inflammation and metabolic dysfunctions,^51^^,^^53^^,^^54^ also declined consistently under both mucin-enriched conditions (Figure 5A,C; qval < 0,0001).

These shifts suggest that mucin availability influences the abundance of key microbial taxa in a niche- and context-dependent manner, with potential implications for host–microbiota interactions. Hence, these results highlight that the mucus compartment selectively shapes the microbiota composition in a donor-specific manner, with consistent enrichment of certain taxa such as *Lachnospirales*. *Roseburia or Faecalibacterium*. The observed variability between donor importantly highlights the suitability of the MBRA 3.0 system to study inter-individual variations in mucus-associated microbial communities.

### The mucus niche impacts microbial metabolism of short-chain fatty acids

Short-chain fatty acids (SCFAs) are key mediators of host–microbiota interactions, with well-documented benefits for host health.^55^^,^^56^ SCFAs are produced by microbial fermentation of substrates such as mucus, and their presence has been shown to upregulate Muc2, the primary mucin in the colon.^57^^,^^58^ While this SCFA-to-mucin feedback loop is well established, the reverse; how mucus availability influences SCFA production has been less extensively studied. A few studies, particularly those involving *A. muciniphila*, suggest that the presence of mucus can enhance the growth of mucin-degrading bacteria and promote SCFAs synthesis.^55^ In light of this, we sought to determine whether the presence of mucus in our MBRA system led to temporal changes in SCFA concentrations.

**Figure 6.**
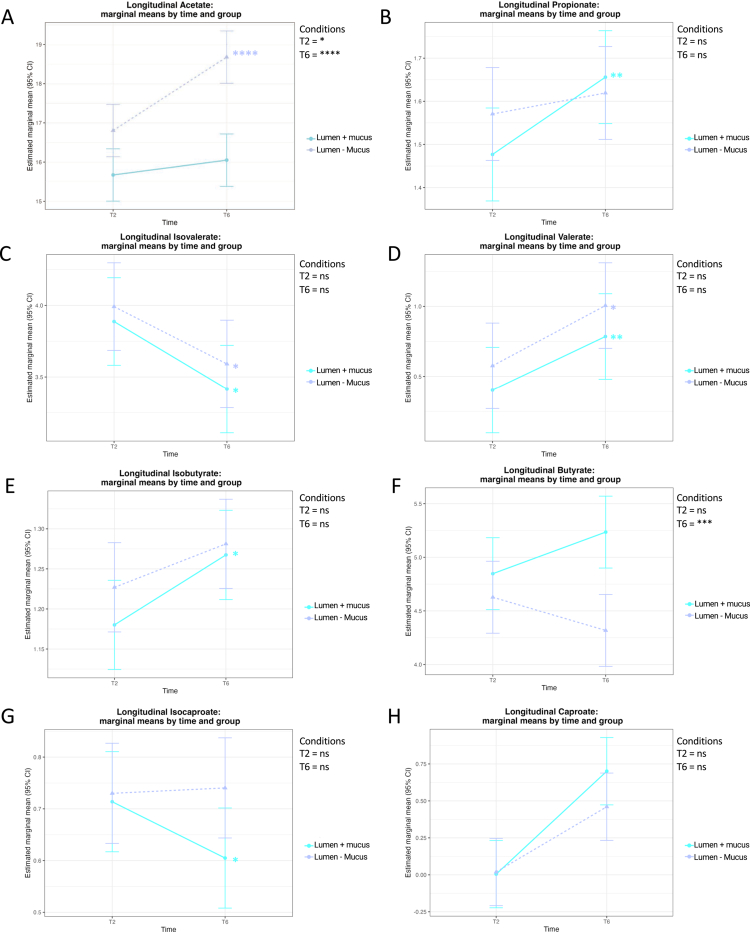
Time-dependent SCFA variation between the lumen–mucus and lumen + mucus. A–H Estimated marginal means (±95%Cis) from linear mixed-effect models (LMMs) are shown for SCFA concentrations measured at T2 and T6 between the two luminal conditions from all the donors (*N* = 8), in the absence (lumen – mucus, dashed purple line) or presence (lumen + mucus, cyan line) of mucus-coated carriers in the bioreactors. Panels represent A. acetate, B. propionate, C. isovalerate, D. valerate, E. isobutyrate, F. butyrate, G. isocaproate, and H. caproate. The significance of the group differences at each timepoint is indicated at the top right corner of the panels, and the intragroup differences between the two timepoints within panels, as follow: ns, not significant; *p ≤ 0,05; **p ≤ 0,01; ***p ≤ 0,001; ****p ≤ 0,0001. Statistical analysis have been assessed using LMMs fitted with donors as a random effect; post hoc pairwise comparisons were corrected using Holm's method; and model residuals distributional assumptions were assessed with DHARMa.

To explore this, we monitored the levels of individual SCFAs at two different timepoints (days 2 and 6) in the lumen with non-coated mucus-carriers (lumen – mucus) and in the lumen containing mucus-coated carriers (lumen + mucus). We first analyzed cohort-level changes using linear mixed-effects models with the donor as a random factor (Figure 6). Overall, the introduction of mucus carriers led to selective and time-dependent alterations in SCFA production. Acetate production increased significantly over time under both conditions; however, the magnitude of this increase was consistently higher in the presence of mucus carriers. This effect was already detectable at T2 (*p* < 0.05) and became strongly significant by T6 (*p* < 0.0001), indicating an enhanced acetate-producing capacity when a mucus niche was provided (Figure 6A). Propionate levels also increased modestly over time but did not differ significantly between conditions (Figure 6B). In contrast, isovalerate and isobutyrate showed a decreasing trend over time under both conditions, without significant condition-dependent differences (Figure 6C,E,G). The valerate and caproate concentrations similarly increased over time but remained comparable between the lumen – mucus and lumen + mucus environments (Figure 6D,H). Among the major fermentation products, butyrate displayed a distinct pattern: while no early differences were observed, a significant divergence emerged at T6, with higher butyrate levels in the lumen + mucus condition (*p* < 0.001; Figure 6F), importantly suggesting that the mucus niche selectively promotes butyrate-producing taxa or enhances their metabolic output at later timepoints.

**Figure 7.**
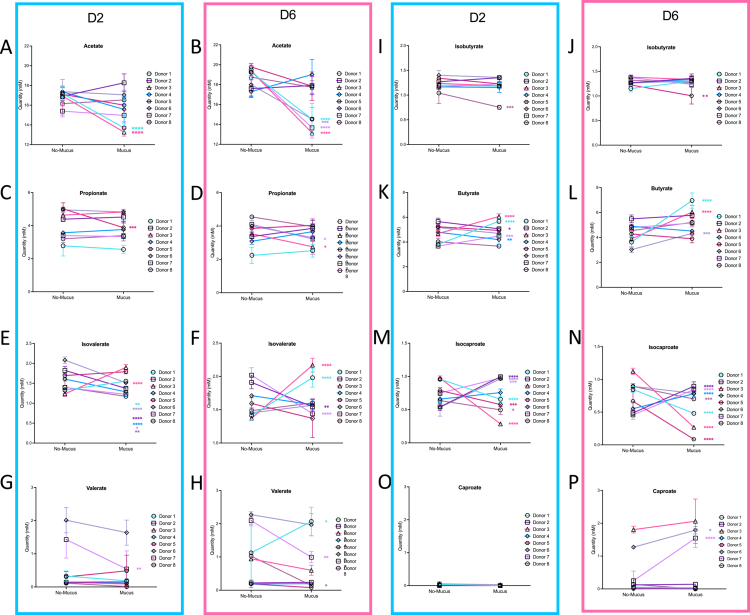
Luminal short-chain fatty acid quantification. Concentrations of major SCFAs – acetate, propionate, butyrate, isobutyrate, isovalerate, valerate, isocaproate, and caproate – measured in luminal samples from eight donors in the absence (no-mucus) or presence (mucus) of mucus-coated carriers. The data are shown for day 2 (left, blue panel) and day 6 (right, pink panels). Each line and symbol represents one donor. The data are expressed as mean + /− S.E.M. for each condition. Significance was assessed by one-way ANOVA and is indicated as follow: *p ≤ 0.05; **p ≤ 0.01; ***p ≤ 0.001; ****p ≤ 0.0001.

Given the donor-dependent variability observed in microbial composition, we next investigated SCFA profiles on a per-donor basis to identify individualized metabolic responses to the presence of mucus carriers (Figure 7). Regarding acetate, the most abundant SCFA in the colon, levels were comparable across all donors in the lumen – mucus condition at both day 2 (D2) and day 6 (D6) (Figure 7A,B). In contrast, in the lumen + mucus condition, a reduction in acetate levels was observed as early as D2 for donors 1, 3, 4, and 8, with a more pronounced decrease at D6 for donors 1, 3, 6, and 7 (Figure 7A,B). For propionate, no notable differences were observed between the lumen – mucus and lumen + mucus conditions at either time point (Figure 7C,D). With respect to isovalerate, a decrease was observed at D2 for all donors except for donors 1, 3, and 5, who instead harbored an increase in the lumen + mucus condition relative to the lumen – mucus (Figure 7E,F), and a similar trend was maintained at D6. Valerate levels slightly decreased in donors 6 and 7 at both D2 and D6, while remaining stable or unchanged in other donors (Figure 7G,H). Butyrate showed a slight increase only in donors 1 and 3 in the lumen + mucus condition (Figure 7K,L). These results highlight the fact that this newly developed MBRA 3.0 system allows the preservation of inter-individual variations in microbiota fermentative capacity, with mucus influencing metabolite production in a donor-dependent manner.

### The mucus niche impacts microbial-derived antigens production

**Figure 8.**
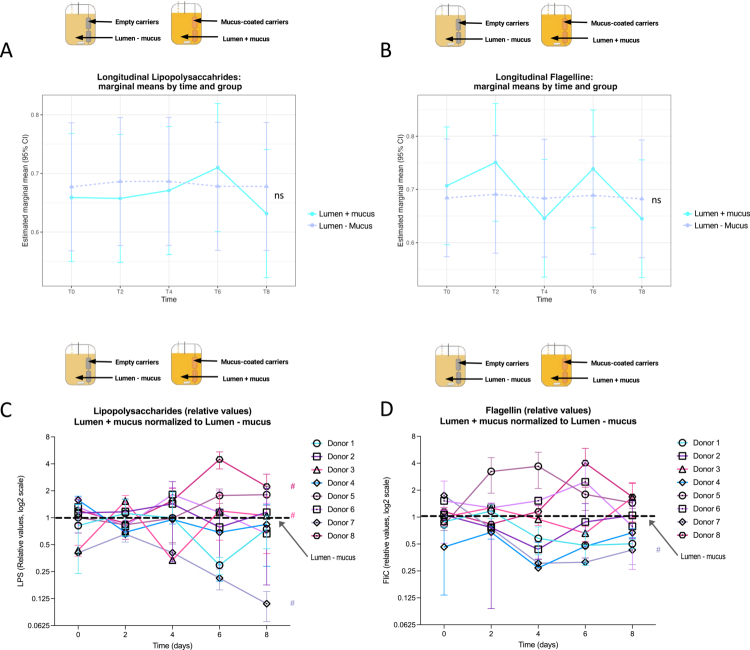
Mucus presence modulates microbiota pro-inflammatory potential overtime in a donor-dependent manner. Estimated marginal means (±95%Cis) from linear mixed-effect models (LMMs) are presented for Lipopolysaccharides A. and Flagellin B. concentrations measured across the experiment for the two luminal conditions from all the donors (*N* = 8), in the absence (Lumen – mucus, dashed purple line) or presence (lumen + mucus, cyan line) of mucus-coated carriers in the bioreactors. Statistical analysis have been assessed using LMMs fitted with donors as a random effect; post hoc pairwise comparisons were corrected using Holm's method; model residuals distributional assumptions were assessed with DHARMa and significance is shown as follow: ns, not significant; *p ≤ 0,05; **p ≤ 0,01; ***p ≤ 0,001; ****p ≤ 0,0001. C. Longitudinal quantification of the bioactive levels of lipopolysaccharide (LPS) in the lumen + mucus microbiota. The data were normalized to the lumen – mucus microbiota, defined as 1 for each donor (*N* = 8). D. Longitudinal quantification of the bioactive levels of flagellin (FliC) in the lumen + mucus microbiota. The data were normalized to the lumen mucus microbiota, defined as 1 for each donor (*N* = 8). The data are expressed as mean + /− S.E.M. for each condition. Significance was assessed by two-way ANOVA and is indicated as follows: # if for at least at one timepoint *p* ≤ 0.05.

Lipopolysaccharides (LPS) and bioactive flagellin (FliC) are the two main pro-inflammatory molecules produced by complex microbiota.^36^ Many factors, including a high-fat diet^5^ and food additives such as emulsifiers,^59^^,^^60^ can modulate LPS and FliC expression by microbiota members. Moreover, we and others previously reported that such levels of microbiota-derived LPS and FliC are good proxy of microbiota “pro-inflammatory potential”, with the observations that such bioactive levels are increased under disease conditions, with levels correlating with disease severity, together with the finding that targeted modulation of such LPS and FliC levels is sufficient to prevent chronic intestinal inflammation.^61-63^ Given the relevance of these two microbiota-derived molecules as intestinal inflammation key drivers, we next assessed whether mucus-carriers modulates their luminal levels. We observed, based on cohort-level analysis using the LMM with the donor as a random effect, that there were no significant differences between the lumen – mucus and lumen + mucus conditions overtime indicating that the presence of mucus does not uniformly shift the LPS or FliC levels in the luminal compartment (Figure 8A,B). In contrast, donor-resolved analyses showed inter-individual variations that were not apparent at the cohort level (Figure 8C,D; Figure S6). In response to mucus addition, luminal LPS levels decreased in donors 1, 4, and 7 while it increased in donors 5 and 8 (Figure 8A). With respect to flagellin, donors 1 and 7 showed a reduction in FliC levels, while donors 6 and 8 exhibited an initial increase followed by a decrease. Other donors showed minimal changes across conditions and time (Figure 8B).

Collectively, these data indicate that the presence of mucus in the MBRA 3.0 reactors impacts the pro-inflammatory potential of the microbiota in a donor-dependent manner. These observations, together with the high-throughput capacity of the MBRA system, importantly suggest that the MBRA3.0 system could be used in future study to better decipher the intricate effect of the mucus layer on functional aspects of the intestinal microbiota, together with a better understanding of inter-individual variations in such interactions.

## Discussion

Over the last two decades, research into the intestinal microbiota–mucus interface has shifted our understanding of the intestinal mucus layer from a static, passive shield to an active, highly specialized ecological niche. Rather than serving solely as a physical blockade, the mucus actively orchestrates microbial colonization, shapes metabolic interactions, and powerfully modulates host immune responses.^64^^,^^65^ The integrity and organization of this interface are now recognized as central for intestinal health, and its disruption is increasingly implicated in the onset and progression of a range of chronic diseases.

The spatial compartmentalization provided by the mucus is indeed critical: it restricts direct microbial contact with the epithelium while simultaneously hosting a unique set of microbes capable of metabolizing mucin glycans. Disruptions in this stratified arrangement, for example, during microbiota encroachment, in which luminal bacteria normally colonize the inner mucus layer, have recently emerged as signature features of chronic pathologies, including inflammatory bowel disease (IBD), metabolic syndrome, both in experimental models and human cohorts.^8-10^^,^^28^^,^^66^ In addition to barrier function, the colonic mucus layer also acts as a dynamic rheostat modulating interactions between the gut microbiota and dietary factors. Several studies have demonstrated that dietary fiber deprivation leads to a rapid depletion of fermentable substrates, pushing microbiota to shift their metabolism towards mucin degradation.^28^ This diet-induced modification results in the proliferation of mucolytic and potentially aggressive bacteria, thinning the mucus barrier and predisposing the host to pathogenic invasion and inflammation. Thus, the mucus layer is not merely a static defense, but rather an active regulator at the interface between diet, microbial ecology, and host health, with its integrity directly shaped by, and responsive to, nutritional availability.^28^

Notably, microbiota encroachment is not a mere byproduct of disease but may act as a causal factor, with recent studies reporting that transferring a mucus-penetrating microbial community from encroachment model mice into germ-free recipients is sufficient to trigger chronic intestinal inflammation.^11^ Further highlighting the central role played by the mucus-associated bacterial population in intestinal and metabolic health, previous studies using targeted immunization of bacterial factors important for mucus colonization by pathobiont members of the intestinal microbiota reported that mucus decolonization is sufficient to protect against chronic intestinal inflammation as well as various aspects of metabolic deregulations. Hence, these insights underscore the need for efficient *in vitro* systems that can both separate and interrogate these spatially distinct but functionally interdependent microbial niches.

Despite the development of various *in vitro* microbiota fermentation models allowing the study of microbiota composition and functions along the different longitudinal parts of the gastrointestinal tract, such as the SHIME,^15^ PolyFermS,^17^ ARCOL,^16^ and TIM-2,^18^ most of these *in vitro* intestinal fermentation models fail to incorporate a mucosal phase, thus limiting our ability to dissect the consequences of mucus‒microbiota separation or encroachment. Systems capable of modeling mucus‒microbiota interactions, such as M-SHIME,^20^ M-ARCOL,^23^ and TIM-2,^18^ are relatively low-throughput and costly and hence are not suited for parallel multi-donor comparative studies, which are now required for multiple dietary factors and/or donors screening. The MBRA platform, in contrast, offers high-throughput, scalable, and flexible continuous anaerobic culturing, which are key features for comparative and personalized microbiome work. Its modular architecture comprises 24 independent chambers per system, and up to six systems can be run in parallel, enabling the simultaneous operation of 144 bioreactors. This configuration supports a wide range of experimental designs, whether cultivating 144 distinct microbiomes, exposing a single community to 144 different compounds, or deploying intermediate layouts tailored to specific research questions. The media delivery system is equally adaptable, with a single reservoir capable of supplying from 1 to 24 chambers, allowing precise adjustment of the experimental scale without altering system performance. Together, these features make the MBRA platform uniquely suited for multiplexed screening, comparative studies, and customizable workflows integrating diverse sample types or treatment conditions. Classic MBRA and its first derivatives, however, were restricted to modeling only the luminal phase,^67^ omitting the mucus compartment and the essential ecological interactions it supports. Owing to the development of the MBRA 3.0, the integration of mucus-coated carriers reconstructs the critical mucosal interface, enabling precise, scalable investigations of mucus-associated community structure and function under controlled and parallelized conditions.

Our comparative analysis of fecal microbiotas from eight healthy donors – selected to represent a range of mucin-degrader abundances – reports that the addition of the mucus niche into the MBRA 3.0 consistently enriches for taxa known to associate with mucin and supports the emergence of a compositionally unique, stable mucus-associated community without disrupting overall luminal bacterial density. The presence of mucus carriers in the system contributed to microbiota stabilization and colonization of the mucus compartment. This observation is consistent with *in vivo* studies showing that mucus provides a structural and nutritional habitat for selected microbiota members.^5^^,^^68^ Additionally, this spatial distinction remains robust across donors, despite substantial inter-individual differences in baseline community structure. Importantly, the addition of mucus did not compromise the system's ability to maintain donor-specific luminal communities, while it enabled the emergence of compositionally distinct mucus-associated ecosystems. Our results highlight that the introduction of mucus carriers significantly influences the luminal community composition in both a donor-dependent and mucus-dependent manner. This highlights that microbial repartition in the gut is a key determinant of community assembly and stability. The selective enrichment of taxa such as *Lachnospirales, Roseburia and Faecalibacterium* within the mucus niche further suggests that mucin availability acts as a targeted ecological filter. While enriched taxa were broadly consistent across donors, the magnitude and direction in which they shifted varied, thus reflecting the inter-individual variability of mucin-degrading taxa. Moreover, we report that the presence of mucus alters not only taxonomic profiles but also microbial function, modulating metabolic outputs, particularly short-chain fatty acid (SCFA) production, as well as the microbiota's pro-inflammatory potential in a donor-dependent manner. SCFA dynamics were comparable across conditions and the presence of mucus consistently increased acetate production throughout the experiment and enhance butyrate levels at later stages, suggesting the selective enrichment of butyrate producers or temporally regulated in cross-feeding networks. These observations reinforce the emerging view that mucosal habitat are metabolically specialized and may serve as key health-associated metabolites production.^5^^,^^48^^,^^69^ Finally, donor-dependent changes in LPS and FliC levels revealed that mucus carriers influence the microbiota's in an individual-dependent manner. The absence of cohort-level effect suggests that the interplay between microbial spatial organization and microbial immunomodulatory molecule production is highly personalized and that the heterogeneity between donors, highlight the MBRA3.0 capacity to uncover individualized metabolic and immunomodulatory signatures. These findings suggest that spatial context is a key variable shaping both compositional and functional outputs of the intestinal microbial ecosystem – an aspect routinely neglected in simple luminal batches.

Furthermore, the MBRA 3.0's throughput and reproducibility unlock new possibilities for systematic and comparative studies. For example, the donor-dependent variability observed here could facilitate dissection of host–microbiota interactions, personalized responses to dietary or pharmacological interventions, as well as the identification of microbial taxa or metabolic patterns that drive or protect against mucus–barrier disruption and disease. Our ability to stably maintain both mucus-associated and adjacent luminal sub-communities (Figure S1) provides a powerful platform for probing the mechanisms underlying encroachment and its role in pathogenesis, for preclinical screening of muco-interactive therapeutics, as well as for the rational design of next-generation probiotics targeted to the mucosal niche that cannot be captured in traditional luminal-only models.

Looking forward, MBRA 3.0 may be further refined to incorporate other features of the *in vivo* mucosal environment, such as dynamic pH gradients, host cell layers, or immune components. Nevertheless, it already represents a major advance in *in vitro* modeling of the spatial structure and functional diversity of the human gut microbiome.

To conclude, by faithfully recapitulating the physical separation and interaction between the mucus and the microbiota, the MBRA 3.0 system opens new avenues for mechanistic research into mucus-related diseases, the testing of muco-active therapies, and the study of how environmental or dietary factors shape health at the mucosal surface. Our results underscore the necessity of integrating spatial ecology into *in vitro* gut models to achieve host-relevant insights and translational discoveries.

## Materials and methods

### Fecal sample collection

Fecal samples were retrieved from an observational clinical study performed at Hôpital Privé des Peupliers (Paris, France), approved by the ethics committee CPP Ile de France VIII (approval number: 210648 and ^70^). Briefly, fresh fecal samples from healthy control without any known diseases or comorbidities (lean, BMI [18,6–27,4]) were collected from participants at their home in a sealed, airtight container with an Anaerocult® system (bioMérieux, Paris, France) to maintain anaerobic conditions. The samples were then transported on ice and delivered within two hours of production in an anaerobic chamber. Aliquots were prepared for various downstream analyses and stored at −80 °C until further use.

### MBRA 3.0 setup

The original MBRA system, as previously described,^20^^,^^21^ consists of 24 individual reactors housed within an anaerobic chamber, each containing 15 mL of BRM, as presented Figure 1A. The reactors were mounted on a magnetic stirrer plate for continuous homogenization and connected to two 24-channel peristaltic pumps (205S, Watson-Marlow) with low-flow capability. To accommodate the experimental requirements and enable the integration of mucus-coated carriers, adjustments were made to the reactors to optimize the operational parameters, including an inversion between the outlet and sample collection holes in order to enlarge the sample collection port to fit a larger adapter (Male Luer 1/8-27" Fitting TSD931-54C, Adhesive Dispensing) and allowing the passage of mucus-coated carriers (Moving Bed Biofilm Reactor (MBBR) P08). In addition, the output PTFE tubing was shortened from 2.5  cm to 1.6  cm to increase the reactor volume to 20 mL and ensure complete immersion of both immersed mucus carriers (Figure 1B). Following autoclaving, the MBRA reactors, tubing, and BRM medium were placed in anaerobic chambers for at least 72 h. The MBRA reactors were then filled with 20 mL of BRM and inoculated with the fecal sample. For inoculation, the fecal samples were resuspended at 2.5% w/v in anaerobic phosphate-buffered saline (D PBS) (Gibco-Life Technologies) within the anaerobic chamber, vortexed for 5 min, and centrifuged at 800  rpm for 5 min at 20 °C. The supernatants were collected in the anaerobic chamber and filtered through a 100-µm filter to remove particles. Each reactor received 1 mL of the fecal slurry. Following the inoculation and installation of mucin carriers, with or without mucin, the fecal microbial communities were allowed to equilibrate for 20 h before initiating flow at 1.875  mL/h (8-h retention time), as illustrated in Figure 1B. Samples (400 µL) were collected over time, and mucin carriers were collected after 48 h spent inside the reactor and stored at −80 °C until further analysis.

### Mucus-coated carriers' preparation and integration in the MBRA 3.0 system

Mucus-coated carriers were incorporated into the system to simulate mucus-associated microbial environments. To mimic colonic mucus turnover, the carriers were replaced every 2 d (Figure 1C). Carriers (Moving Bed Biofilm Reactor [MBBR], P08) were sterilized by immersion in absolute ethanol for 24 h, followed by air drying under sterile conditions. Carriers were then coated with a mucin–agar matrix, as previously described.^22^ Briefly, sterile water was boiled, and 1% (w/v) agar and 5% (w/v) porcine mucin type II (Prodigest, Ghent, Belgium) were added under continuous agitation. The mixture was homogenized, and the pH was adjusted to 6.8 using 10 M NaOH before coating the carriers (Figure 1E).

### DNA extraction

Bacterial DNA was extracted from 50 µL of frozen MBRA suspension or 100 µL of mucin recovered from mucus-coated carriers suspended in dPBS. Extraction was performed using the DNeasy 96 PowerSoil Pro QIAcube HT Kit (Qiagen), with mechanical disruption via bead beating (TissueLyzer II, Qiagen). In brief, the samples were transferred to 96-well PowerBead plates and mixed with 800 µL of CD1 lysis buffer. The cells were lysed by bead beating for 5 min using the TissueLyzer II to release DNA. The plates were then centrifuged at 2,773 × g for 10 min at 20 °C to pellet the debris and beads. A volume of 500 µL of the supernatant was transferred to an S-block (96-well plate), mixed with CD2 solution, and centrifuged again under the same conditions to remove proteins and inhibitors. Next, 550 µL of the resulting supernatants were transferred to a new S-block, and the following steps were carried out on the QIAcube HT platform: 450 µL of C3 binding buffer was added to facilitate DNA binding to the QIAamp 96-well silica membrane plates. The bound DNA was washed sequentially with 1 mL of AW1 buffer, 600 µL of AW2 buffer, and 400 µL of 96% ethanol. DNA was eluted in a total volume of 150 µL using a combination of 120 µL of C6 and 30 µL of Top Elute buffer and stored at −20 °C until further analysis.

### Bacterial load quantification through 16S rRNA qPCR

Extracted DNA was diluted tenfold in sterile, DNA-free water prior to quantitative PCR (qPCR) analysis targeting the V4 region of the bacterial 16S rRNA gene. Amplification was performed using universal primers 515F (5′-GTGYCAGCMGCCGCGGTAA-3′) and 806 R (5′-GGACTACNVGGGTWTCTAAT-3′). Reactions were run on a StepOnePlus™ Real-Time PCR System (Applied Biosystems) using the QuantiFast SYBR® Green PCR Kit (Qiagen) following the manufacturer's protocol.

### Microbiota analysis by 16S rRNA gene sequencing using Illumina technology

Microbial community composition was assessed by 16S rRNA gene amplicon sequencing using the Illumina MiSeq platform. The V4 region of the 16S rRNA gene was amplified via polymerase chain reaction (PCR) from each sample using a composite forward primer and a reverse primer, each incorporating a unique 12-base barcode designed with the Golay error-correcting scheme to enable sample-specific tagging of PCR products. The forward primer 515F used for amplification was as follows:

5’-*AATGATACGGCGACCACCGAGATCTACACGCT*XXXXXXXXXXXX**TATGGTAA TT*****GT***GTGYCAGCMGCCGCGGTAA-3’. The italicized segment corresponds to the 5’ Illumina adapter, the 12 'X' characters represent the Golay barcode, the bold portion denotes the primer pad, the italicized and bold sequence serves as the primer linker, and the underlined section is the conserved bacterial primer 515F. Similarly, the reverse primer 806 R used was:

5’-*CAAGCAGAAGACGGCATACGAGAT***AGTCAGCCAG*****CC***GGACTACNVGGGTWTCTAAT-3’. Here, the italicized sequence represents the 3’ reverse complement of the Illumina adapter, the bold segment corresponds to the primer pad, the italicized and bold sequence functions as the primer linker, and the underlined section is the conserved bacterial primer 806R.

PCR amplification was performed using 5PRIME HotMasterMix (Quantabio, Beverly, MA, USA) with a final primer concentration of 0.2  μM and a template DNA input ranging from 10 to 100 ng. The thermal cycling conditions included an initial denaturation at 95 °C for 3 min, followed by 30 cycles of 95 °C for 45 s, 50 °C for 60 s, and 72 °C for 90 s on a Bio-Rad thermal cycler. Amplicons were subsequently analyzed via agarose gel electrophoresis. DNA quantification was performed using the Quant-iT PicoGreen dsDNA assay, and equimolar amounts of PCR products were pooled to generate a master DNA library, which was further purified using AMPure magnetic beads (Agencourt, Brea, CA, USA) before sequencing (paired-end reads, 2 × 250 bp) on an Illumina MiSeq platform at the Genom'IC facility, Institut Cochin, Paris, France. Unprocessed sequencing data are available in the European Nucleotide Archive under accession number PRJEB95761.

### 16S rRNA gene sequence analysis

The 16S rRNA sequences were analyzed using QIIME2 (version 2022). Sequence demultiplexing and quality filtering were performed using the Dada2 algorithm with default QIIME2 parameters to identify and correct Illumina amplicon sequencing errors and produce an amplicon sequence variant (ASV) table. The following DADA2 command was employed to generate a QIIME2 artifact table using: “qiime dada2 denoise-paired --i-demultiplexed-seqs demux.qza --p-trim-left-f 0 --p-trim-left-r 0 --p-trunc-len-f 180 --p-trunc-len-r 180 --o-representative-sequences rep-seqs-dada2.qza --o-table table-dada2.qza --o-denoising-stats stats-dada2.qza --p-*n*-threads 6” Dada2 command. A phylogenetic tree was subsequently constructed using the align-to-tree-mafft-fasttree pipeline to support phylogenetic diversity calculations. To normalize sequencing depth across samples prior to diversity analyses, the ASV table was rarefied to 5.929 sequences per sample, and samples below this threshold were excluded (Figure S7, Table S1). Alpha and beta diversity metrics were computed using the core-metric phylogenetic workflow, with beta diversity visualized via principal coordinates analysis (PCoA) plots. ASVs were used for all analyses unless taxonomic aggregation was required. For taxonomic bar plots and MaAsLin2 modeling, which require operational taxonomic units (OTUs) rather than ASVs, sequences were clustered into OTUs at 99% identity using Greengenes2 reference database (version 2022.10).

### Quantification of short chain fatty acids production

Short-chain fatty acids (SCFAs) were quantified from MBRA supernatants to assess microbial metabolic activity. A total of 100  µL of frozen MBRA samples were centrifuged at 12,000  rpm for 15  min at 4 °C. The supernatants were collected and sent to the INRAE, Micalis (Jouy-en-Josas, France), for SCFA analysis. Briefly, 10% of phosphotungtic acid was added to each sample to precipitate proteins overnight at 4 °C, followed by centrifugation at 12,000  rpm for 15  min at 4 °C. The supernatants were collected and mixed with 20 mM 2-ethylbutyrate at a 1:4 ratio, which served as an internal standard. SCFA concentrations were determined via gas chromatography (Agilent 7890B, Agilent Technologies, Les Ulis, France). A 0.3 µL injection volume was used, with hydrogen as the carrier gas at a flow rate of 10  mL/min. The GC oven was programmed as follows: initial temperature at 100 °C for 10 min, ramped to 180 °C at 20 °C/min, and held for 2 min. The detector temperature was set to 240 °C. All the samples were analyzed in duplicate. Chromatographic peaks were integrated using OpenLAB ChemStation software (Agilent Technologies, Les Ulis, France) as previously described.^71^

### Quantification of bioactive flagellin and lipopolysaccharide

Bioactive flagellin and lipopolysaccharide (LPS) levels in MBRA supernatants were quantified using Human Embryonic Kidney (HEK)-Blue-mTLR5 and HEK-Blue-mTLR4 reporter cells (InvivoGen, San Diego, California), as previously described.^37^ MBRA supernatants were serially diluted in culture medium and applied to the cells. Standard curves were generated using purified *Salmonella typhimurium* flagellin (FLA-ST, InvivoGen) and *Escherichia coli* O111:B4 LPS (Sigma-Aldrich) for the TLR5 and TLR4 assays, respectively. Following overnight stimulation at 37 °C in a humidified 5% CO_2_ atmosphere, the culture supernatants were transferred to QUANTI-Blue medium (InvivoGen, San Diego, California). Secreted embryonic alkaline phosphatase (SEAP) activity was quantified by measuring the absorbance at 620 nm after 30 min of incubation at 37 °C.

### Statistical analysis

All the statistical analyses were performed in R (version 4.4.1) or GraphPad Prism (version 10.1.1). Depending on the structure of each dataset, analyses were conducted either per donor or at the cohort level with the donor treated as a random effect.

### Longitudinal analyses of bacterial load

For per-donor analyses, longitudinal changes in bacterial load were assessed using two-way ANOVA with time and condition as fixed effects. When all donors were analyzed jointly, a linear mixed-effects model (LMM) was applied with condition and time as fixed effects and donor as a random effect. Residual diagnostics were performed using DHARMa, and Holm correction was applied for post–hoc pairwise comparisons.

### Alpha and beta diversity

For per-donor analyses of alpha and beta diversity metrics, two-way ANOVA with Sidak's correction for multiple comparisons was used.

For cohort-level comparisons, beta diversity distance matrices were evaluated using PERMANOVA (Adonis2), with the donor treated as a random effect via stratification. Group dispersions were tested using PERMDISP, and post–hoc pairwise contrasts were performed using Tukey’s HSD when appropriate.

### SCFA, LPS, and flagellin quantification

When donors were analyzed jointly, SCFA, LPS, and flagellin concentrations were evaluated using linear mixed-effects models with the donor included as a random effect. Residual diagnostics were performed using DHARMa, and Holm correction was applied for post–hoc pairwise comparisons. For per-donor analyses, two-way ANOVA with Sidak's correction for multiple comparisons was used for LPS and flagellin, and one-way ANOVA with Dunnetts's correction for multiple comparisons was used to assess intra-donor variation in SCFAs, LPS, or flagellin across conditions or timepoints.

### Identification of differentially abundant microbiota members

Microbial taxa exhibiting significant differences in relative abundance between experimental conditions were identified using MaAsLin2 (microbiome multivariable associations with linear models, version 2).^72^ Analyses were performed in R (version 4.4.1) using the MaAsLin2 package (version 1.12.0), with input data consisting of genera-level relative abundances. Microbiota members were reported as significantly altered in their relative abundance if corrected *q*-value ≤ 0.05 or *p*-value ≤ 0.05.

### Significance threshold

*p*-values < 0.05 were considered statistically significant.

## Supplementary Material

Supplemental figures.pdf Supplemental figures.pdf

Supplementary table 1_VF2 copy.xlsx Supplementary table 1_VF2 copy.xlsx

Supplementary material Supplementary_file.docx

## Abbreviations

BRM: BioReactor Medium
CAZymes: Carbohydrate Active enZymes
FliC: Flagellin
IBD: Inflammatory Bowel Diseases
LPS: Lipopolysaccharides
LMM: Linear Mixed effect Model
MaAsLin: Microbiome Multivariate Association with Linear Models
MBRA: MiniBioreactor Array
M-SHIME: Mucosal- Simulator of the Human Intestinal Microbiome Ecosystem
MUC2: Mucin 2 gene
SCFA: Short Chain Fatty Acid
