# Supplementary material for: MBRA 3.0: integrating the mucus environment for advanced high-throughput in vitro intestinal microbiome modeling
Source: Gut Microbes. 2026 Jan 11;18(1):2612804. doi: 10.1080/19490976.2026.2612804 (PMC12795259; doi:10.1080/19490976.2026.2612804)
Supplement: Supplemental figures.pdf [file KGMI_A_2612804_SM0752.pdf]

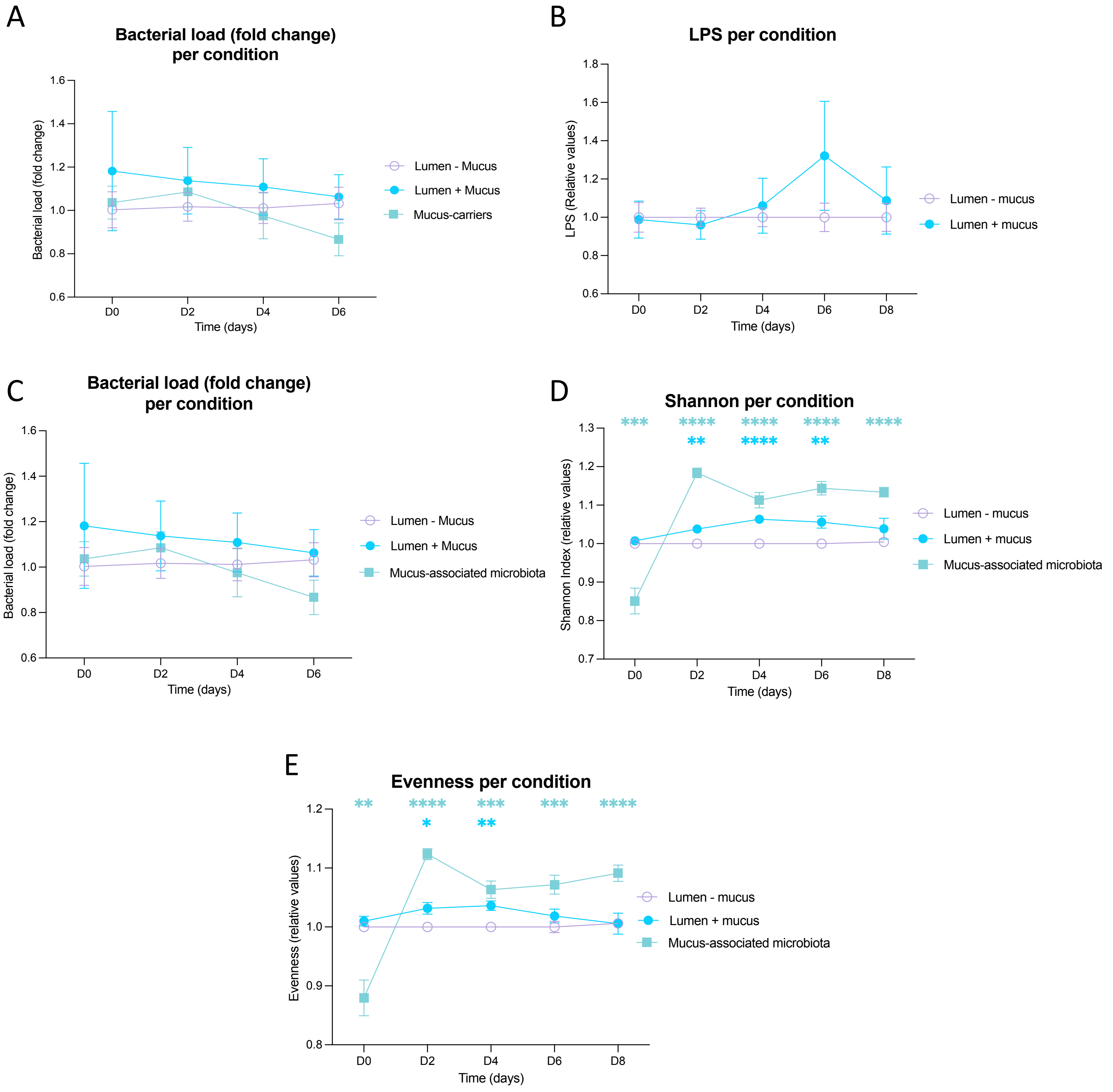

Supplementary figure 1: Longitudinal microbial and diversity profiles across conditions

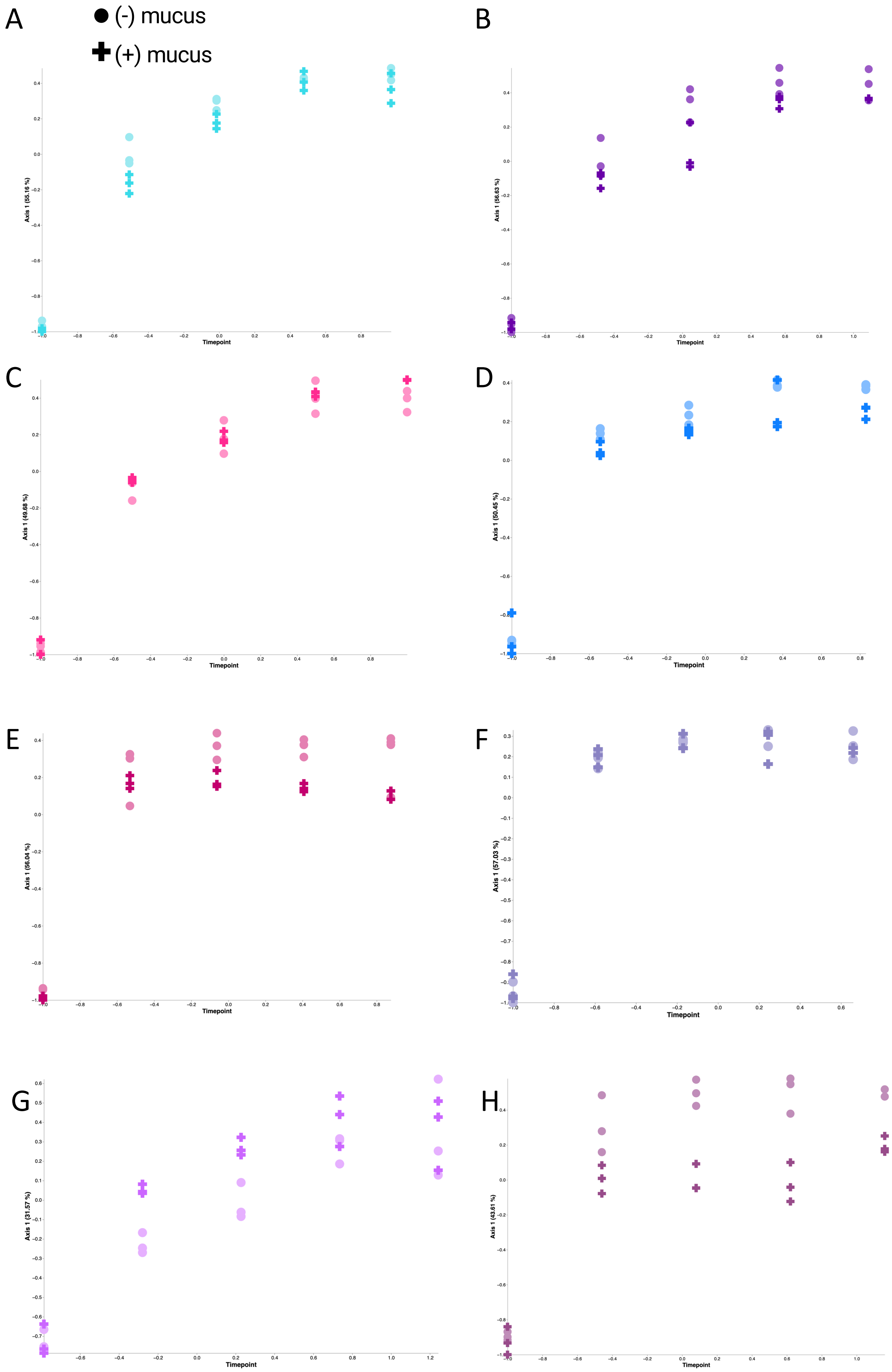

Supplementary figure 2: Temporal intra-individual variation of microbiota composition between lumen – mucus and lumen + mucus ecosystems.

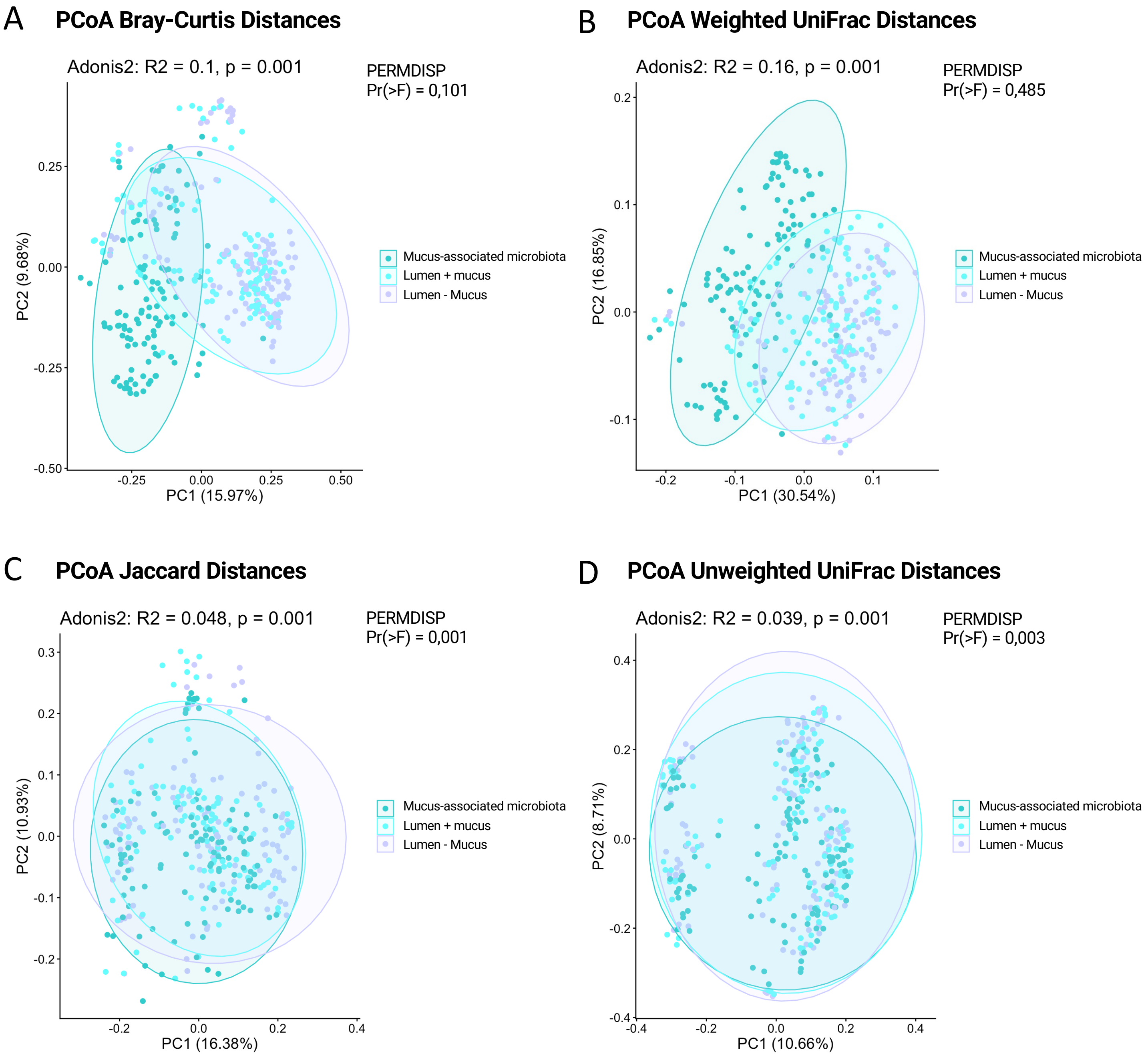

Supplementary figure 3: PCoA of microbial community structure across the different conditions.

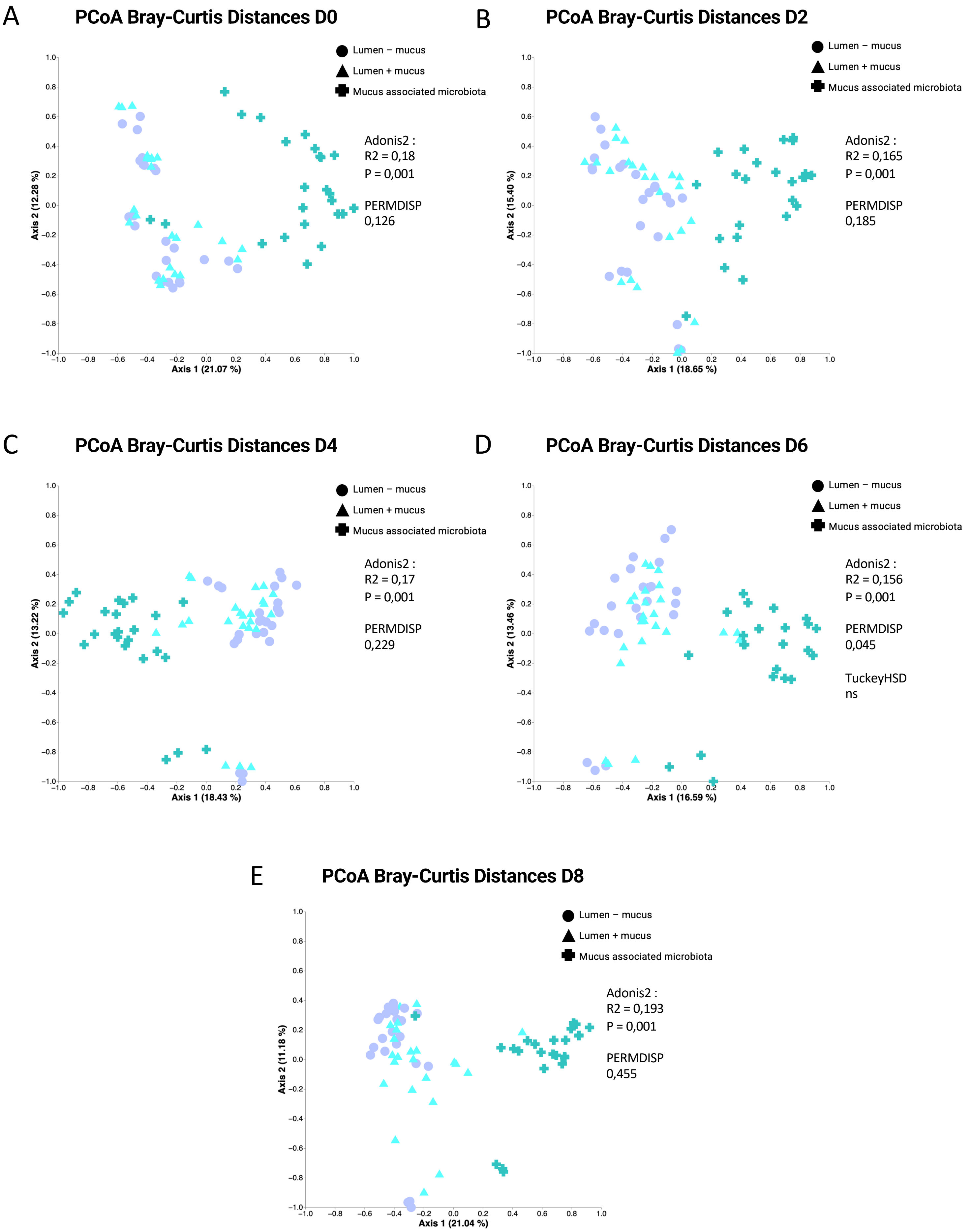

Supplementary figure 4: Temporal evolution of microbial community structure across conditions.

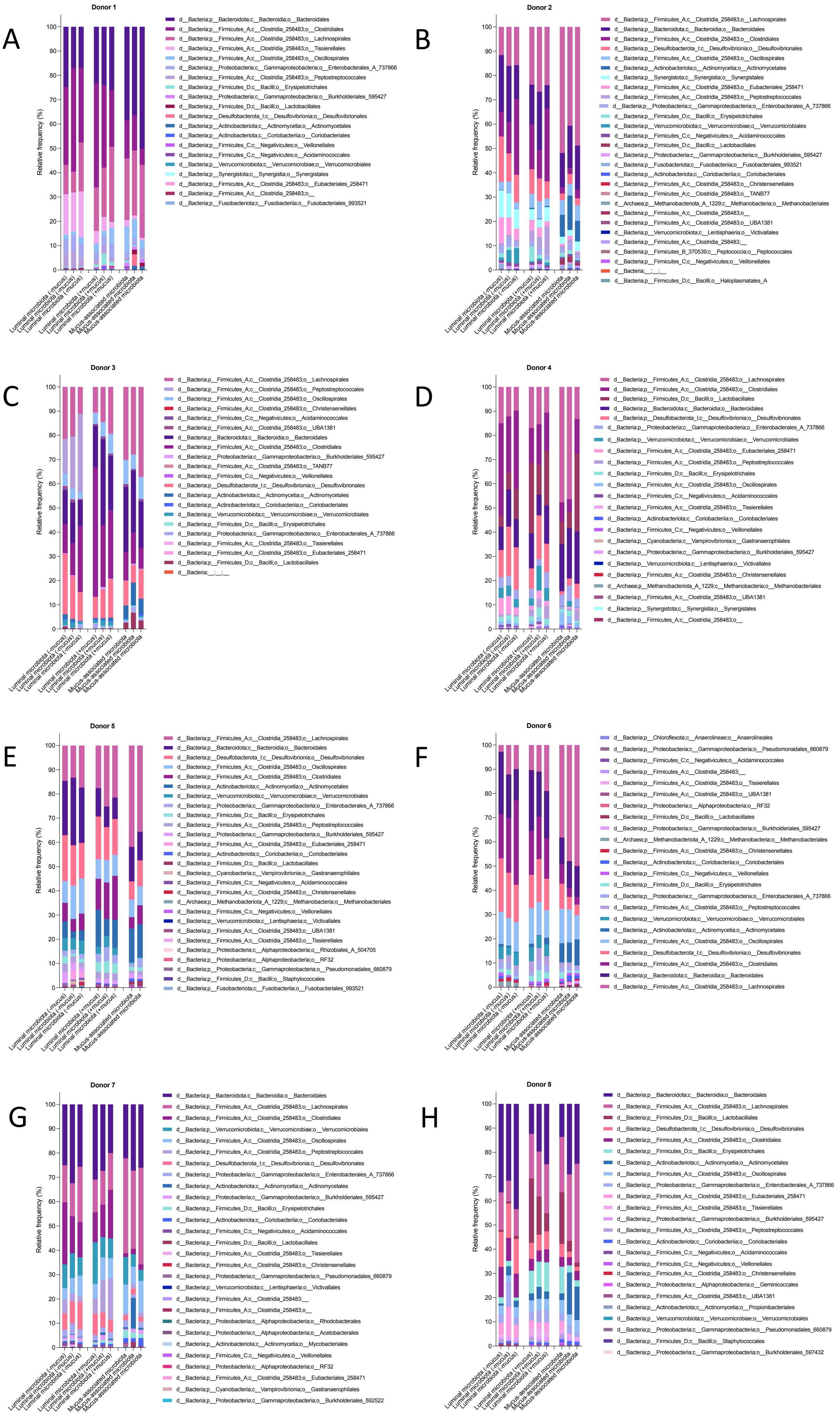

Supplementary figure 5: Taxonomical composition of luminal and mucus-associated microbiota in individual donors.

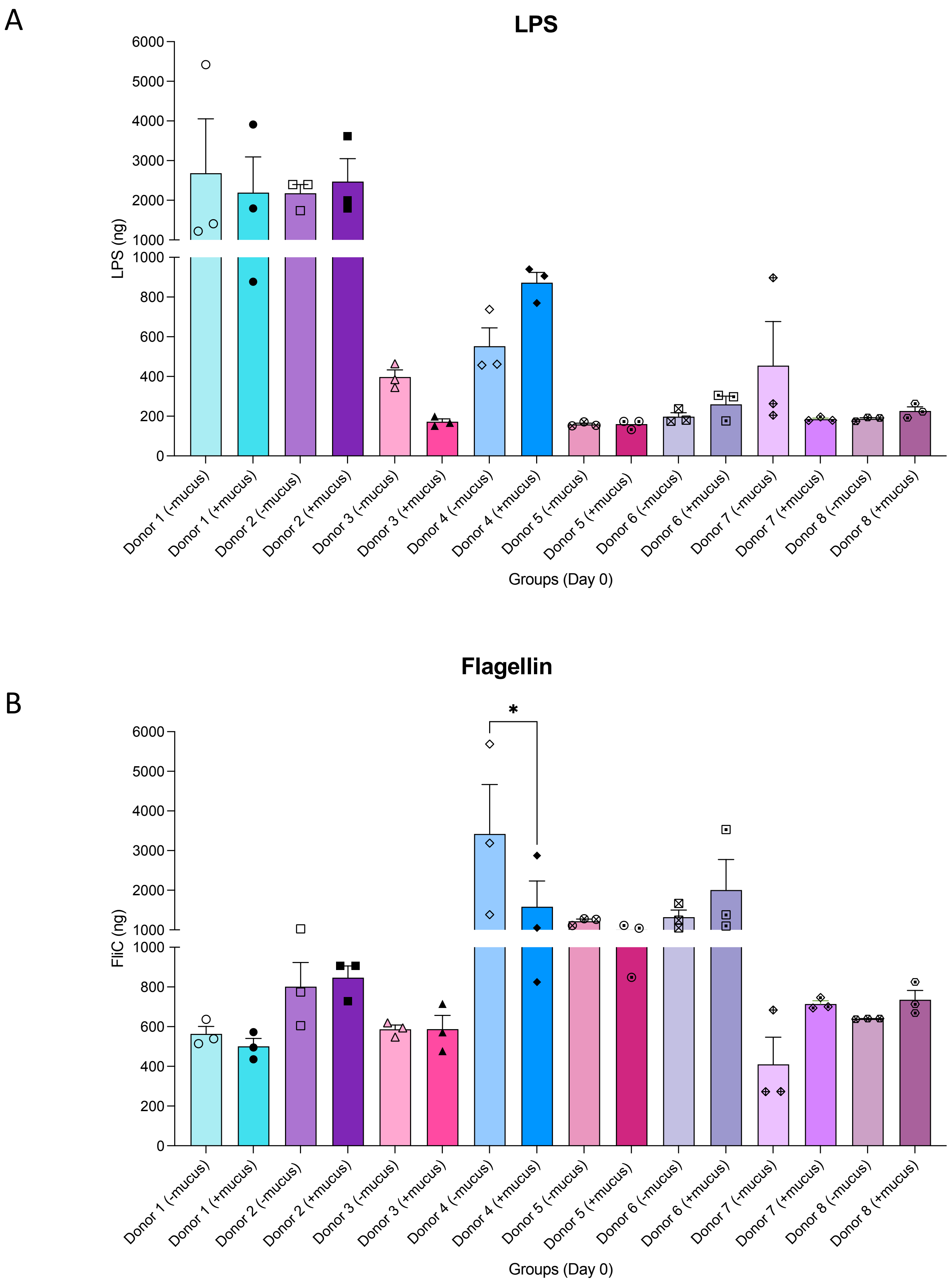

Supplementary figure 6: Intra-donor variability in bioactive levels of LPS and FliC across bioreactor replicates in the presence or absence of mucus at Day 0.

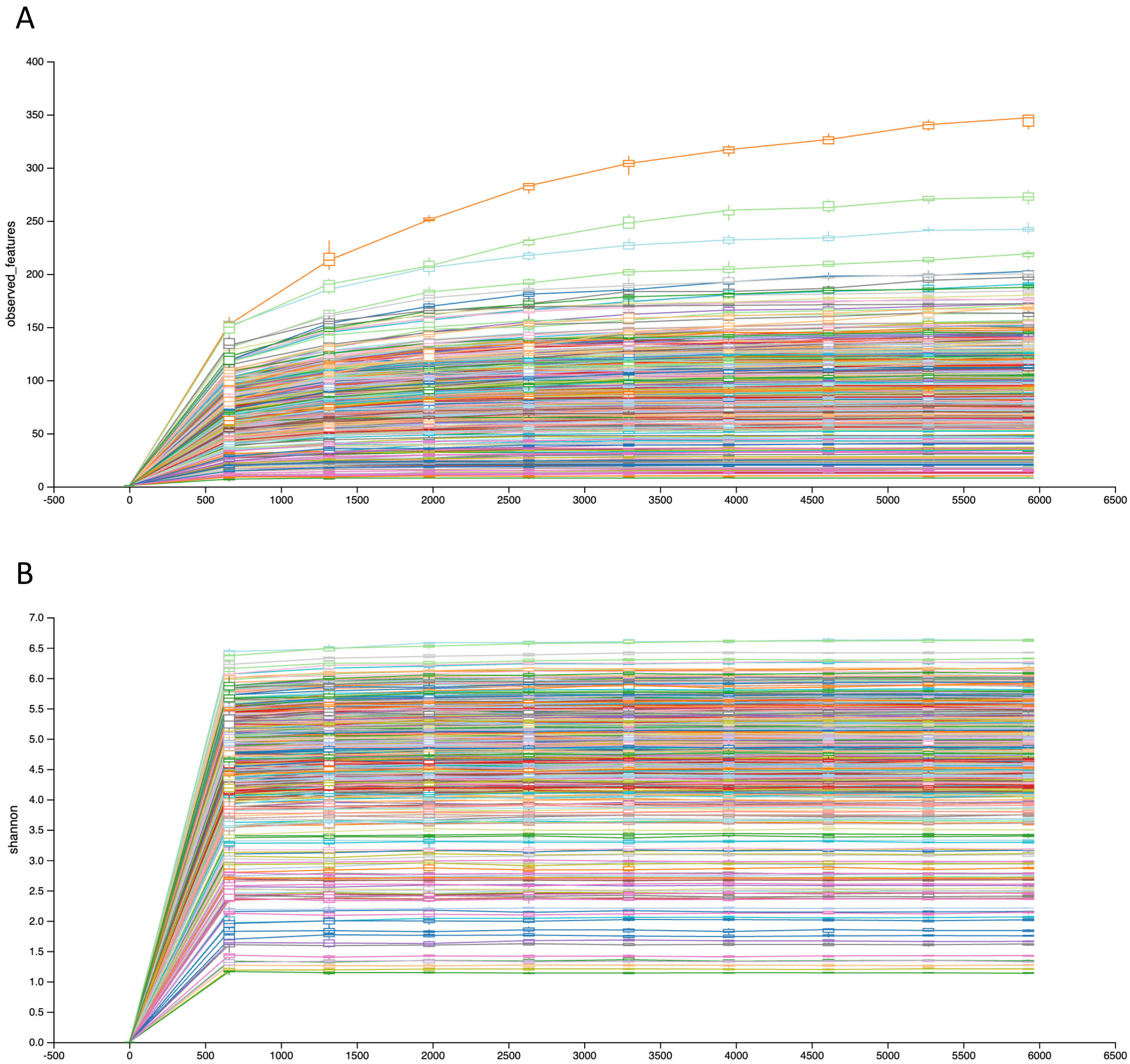

Supplementary figure 7:
