# Supplementary material for: MBRA 3.0: integrating the mucus environment for advanced high-throughput in vitro intestinal microbiome modeling
Source: Gut Microbes. 2026 Jan 11;18(1):2612804. doi: 10.1080/19490976.2026.2612804 (PMC12795259; doi:10.1080/19490976.2026.2612804)
Supplement: Supplementary material — Supplementary_file.docx [file KGMI_A_2612804_SM8787.docx]

**Supplemental figure legends**

**Supplementary figure 1: Longitudinal microbiota analysis across conditions**. **A-** Bacterial load (fold change), **B-** lipopolysaccharide (LPS) levels, **C-** flagellin (FliC) levels, **D-** Shannon diversity index, and **E-** Evenness index measured across the three experimental conditions: **Lumen – mucus** (purple circles and line), **Lumen + mucus** (blue circles and line), and **mucus-associated microbiota** (teal squares and line). Measurements were taken longitudinally. Data represent mean ± S.E.M. Statistical significance was assessed using a **two-way ANOVA** (factors: condition × time), followed by **Tukey’s post hoc test** for pairwise comparisons. Significant differences between conditions at each time point are indicated as follow *p ≤ 0.05,** **p** ≤ 0.01, ******p*** ≤ 0.001, *****p** ≤ 0.0001)

**Supplementary figure 2: Temporal intra-individual variation of microbiota composition between lumen – mucus and lumen + mucus ecosystems*.*** Longitudinal analysis of axis 1 of PCoA of the Bray-Curtis distance for lumen – mucus (circles) and lumen + mucus (crosses) throughout time. Each panel represents a single donor.

**Supplementary figure 3: Principal Coordinates Analysis of microbial community structure across the different mucus conditions A-** Bray–Curtis, **B-** Weighted UniFrac, **C-** Jaccard, and **D-** Unweighted UniFrac distance–based PCoA plots showing beta-diversity differences among the three experimental conditions: Lumen – mucus (purple), Lumen + mucus (blue), and Mucus-associated microbiota (teal). Each point represents one sample, and ellipses indicate 95% confidence intervals for group dispersion. Differences in community structure were assessed using PERMANOVA (Adonis2), with the corresponding R² and p-values. Homogeneity of group dispersions was evaluated using PERMDISP, with p-values displayed accordingly. When PERMDISP indicated significant differences in dispersion, pairwise comparisons were performed using TukeyHSD to identify which groups differed in variance structure.

**Supplementary figure 4:** **Temporal evolution of microbial community structure across conditions (Bray–Curtis PCoA). A–E-** Principal Coordinates Analysis (PCoA) based on Bray–Curtis distances at Day 0 **A-**, Day 2 **B-**, Day 4 **C-**, Day 6 **D-**, and Day 8 **E-** for the three experimental conditions: lumen - mucus (circles), lumen + mucus (triangles), and mucus-associated microbiota (crosses). Each point represents an individual sample. Significance was assessed using PERMANOVA (Adonis2), with the corresponding R² and p-values. Homogeneity of group dispersions was evaluated using PERMDISP, with p-values displayed accordingly. When PERMDISP indicated significant differences in dispersion, pairwise comparisons were performed using TukeyHSD to identify which groups differed in variance structure.

**Supplementary figure 5: Taxonomical composition of luminal and mucus-associated microbiota in individual donors**. Relative abundance of bacterial taxa at the order level in donors 1 to 8 **A-H**. The three distinct compartments are presented: Lumen – mucus, Lumen + mucus and mucus-associated microbiota. Each bar represents a sample collected at D8 in one single bioreactor or a mucus carrier.

**Supplementary figure 6: Intra-donor variability in bioactive levels of LPS and FliC across bioreactor replicates in the presence or absence of mucus at Day 0**. Histogram representation of non-normalized bioactive levels of LPS **A-** and FliC **B-** at day 0 in luminal microbiota samples from the eight individual donors under two conditions: in the in the absence (−mucus) and in the presence (+mucus) of mucus. For each donor, histograms represent mean +/- S.E.M., with individual data points being presented. Donors and conditions are color-coded as indicated. Significance was assessed by one-way ANOVA and is indicated as follow: * p ≤ 0.05.

**Supplementary figure 7: Rarefaction curves of the Illumina-based 16S rRNA sequencing data. A-** Rarefaction curves displaying the number of observed ASVs as a function of sequencing depth for all samples. **B-** Rarefaction curves displaying Shannon diversity values as a function of sequencing depth for all samples. Curves were generated using the QIIME2 alpha-rarefaction method.

**Supplementary table 1: Sequencing and quality-filtering metrics from the Illumina-based 16S rRNA sequencing data.** Summary of sequencing reads, filtering statistics, post-filter sequencing depth, and ASV counts for each sample. The rarefaction depth used in diversity analyses was 5.929 reads.
